# Supplementary material for: The sulfite oxidase Shopper controls neuronal activity by regulating glutamate homeostasis in Drosophila ensheathing glia
Source: Nat Commun. 2018 Aug 29;9:3514. doi: 10.1038/s41467-018-05645-z (PMC6115356; doi:10.1038/s41467-018-05645-z)
Supplement: Supplementary file 1 — Supplementary Information [file 41467_2018_5645_MOESM1_ESM.pdf]

The Sulfite Oxidase Shopper controls neuronal activity by regulating glutamate homeostasis in *Drosophila* ensheathing glia

Supplementary Information

Nils Otto[1#], Zvonimir Marelja[$§2], Andreas Schoofs[$3], Holger Kranenburg [$1], Jonas Bittern[1], Kerem Yildirim[1], Dimitri Berh[4], Maria Bethke[1], Silke Thomas[1], Sandra Rode[1], Benjamin Risse[4], Xiaoyi Jiang[4], Michael Pankratz[3], Silke Leimkühler[2], Christian Klämbt*[1]

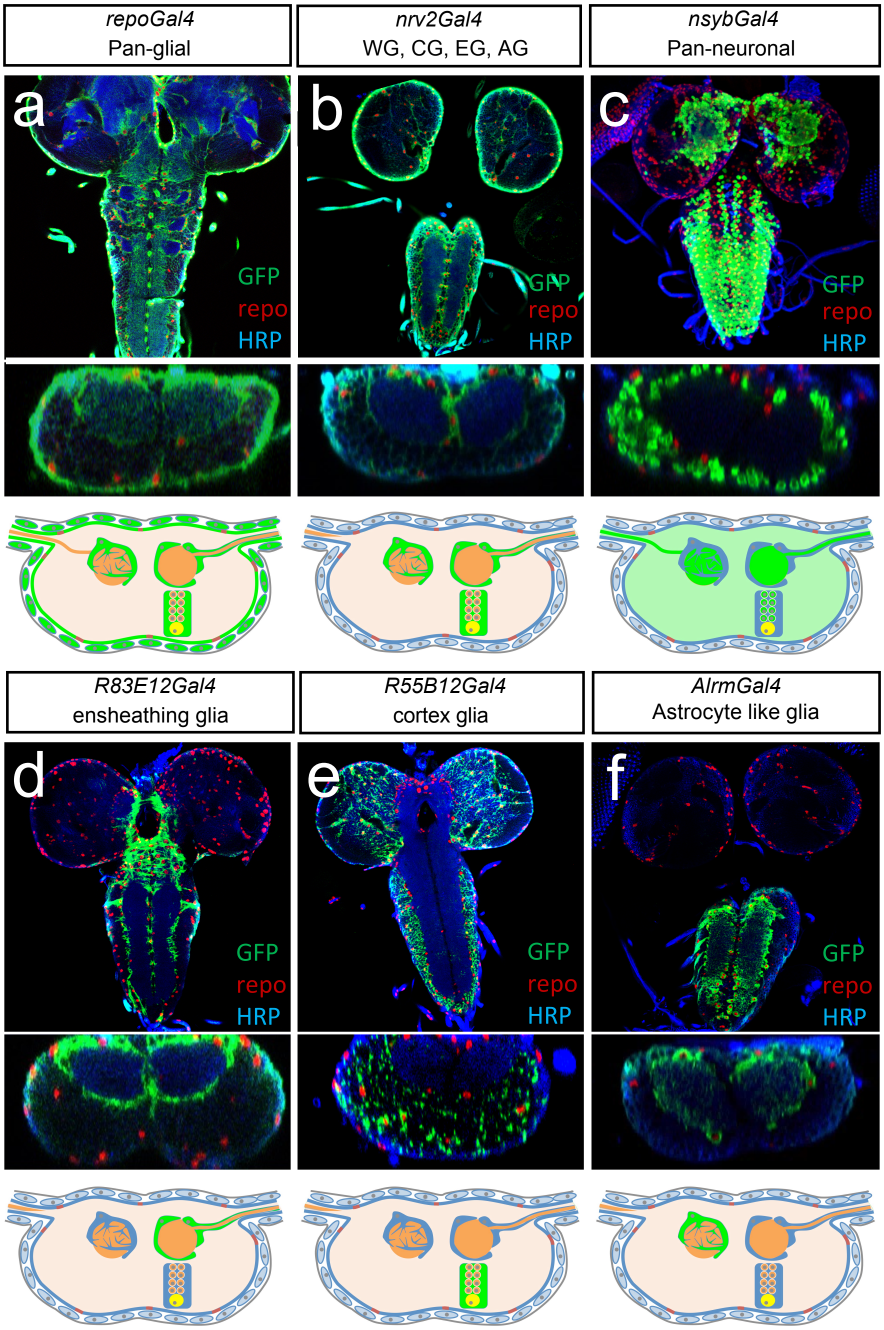

Supplementary Figure 1 Expression patterns of employed drivers

a-f Single confocal sections of third instar larval brains of indicated genotypes stained for GFP (green), the pan-glial nuclear marker Repo (red), and neuronal cell membranes (aHRP, blue). The different glial cells targeted by the respective Gal4 drivers are indicated. The schematic drawing summarizes the expression patterns. For further labeling compare to Fig. 1h. Below each image are representative orthogonal sections of the same brains and schemes indicating the position of targeted cells. **a** *repo-Gal4* directs expression to all glial cells. **b** *nrv2-Gal4* directs expression to wrapping glial cells in the peripheral nervous system (WG), cortex glial cells (CG), ensheathing glial cells (EG), and astrocytes (AG). **c** nsyb-Gal4 directs expression to all neurons. **d** *R83E12-Gal4* directs expression to ensheathing glial cells exclusively. **e** *R55B12-Gal4* directs expression to cortex glial cells exclusively. **f** *alrm-Gal4* directs expression to astrocytes exclusively. Scale bars are 20 µm.

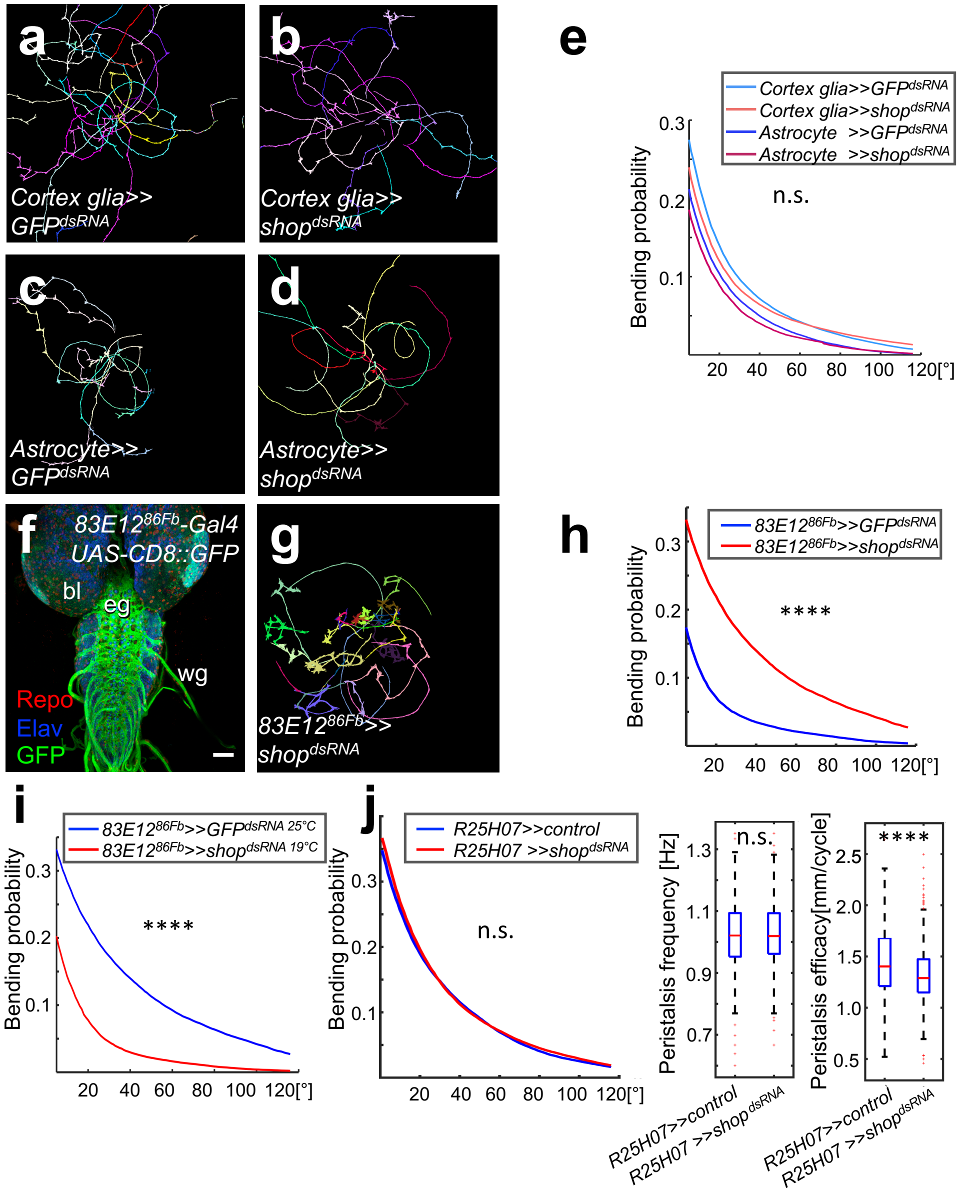

Supplementary Figure 2 Suppression of *shopper* in astrocytes and cortex glia does not change behavior

Cortex glia (*R55B12-Gal4)* and astrocyte specific suppression *(alrm-Gal4 and R25H07-Gal4).* **a** Control animals of the genotype *UAS-GFP^{dsRNA}; R55B12-Gal4 (n=253 trajectories)* do not exhibit a different behavior compared to **b** where *shopper* is suppressed *(n=206 trajectories)*. **c** RNAi control animals of the genotype *alrmGal4; UAS-GFP^{dsRNA}; alrmGal4 (n=116 trajectories)* do not exhibit a different behavior than upon **d** suppression of *shopper (n=145 trajectories)*. **e** Quantification of bending probability of the genotypes indicated. Suppression of *shopper* neither in cortex glia nor in astrocytes causes locomotion defects like described for ensheathing glia. **f** Antibody staining of a third instar larval brain of the genotype: *R83E12^{86Fb}-Gal4*, *UAS-CD8::GFP*. The expression domain of the *Gal4* driver is comparable to the original *R83E12-Gal4* insertion in the *attp2* site (see Supplementary Figure 1). **g,h** Suppression of *shopper* using *R83E12^{86Fb}-Gal4 (n=125 trajectories)* increases head bending compared to controls *(n=87 trajectories)*. **i** *shopper* suppression in ensheathing glia results in a stronger phenotype when tested at 25° C *(n=125 trajectories)* compared to 19°C *(n=74 trajectories)*. **j** Analysis of *shopper* suppression using the astrocyte driver *R25H07-Gal4*. *UAS-shop^{dsRNA}; R25H07-Gal4* animals *(n=79 trajectories)* compared to *UAS-GFP^{dsRNA}; R25H07-Gal4 (n=87 trajectories)* do not show increased bending probability. While peristalsis efficacy between these genotypes *(n=459 run phases vs. 288 run phases)* is reduced, no change in peristalsis frequency can be observed. Scale bars are 20 µm. Box plots represent: The first and third quartile, as well as the median. Whiskers contain values within 1.5x of the interquartile range, outliers are red crosses. >600 frames of ~80 larvae; ****p<0.001, n.s.: not significant, Wilcoxon rank-sum Test. See supplementary Table 1 for details.

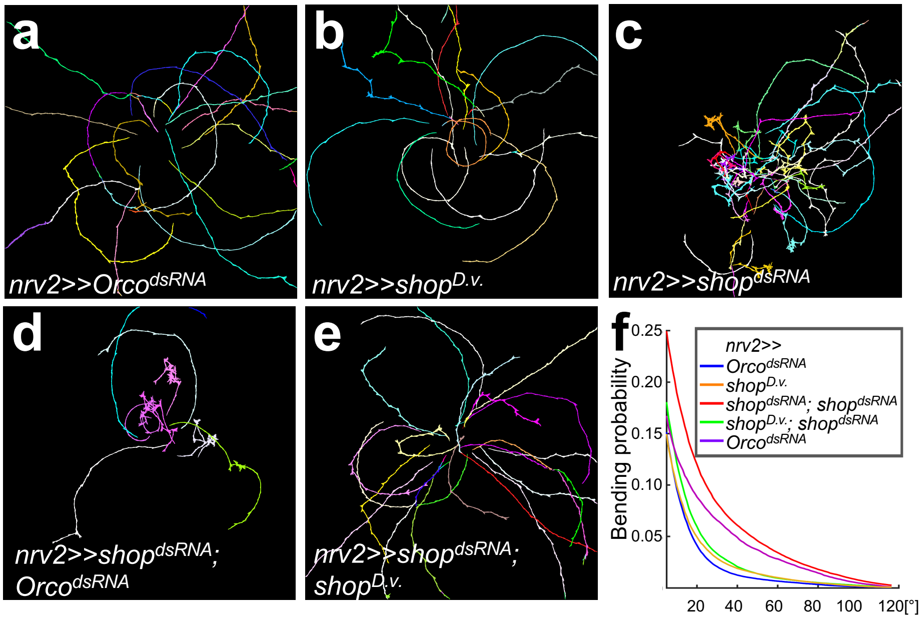

Supplementary Figure 3 The shopper RNAi phenotype can be rescued by an RNAi insensitive interspecies rescue construct

Locomotion features were recorded following expression of different transgenes in ensheathing and cortex glia using *nrv2-Gal4.* **a** RNAi control animals of the genotype *nrv2-Gal4, UAS-Orco*^{dsRNA}*; nrv2-Gal4. Orco* is not expressed by glial cells. **b** Expression of the *D. virilis shopper* gene did not cause alterations from the control. **c** *nrv2-Gal4* driven knockdown of *shopper* causes a head bending phenotype. **d** *nrv2-Gal4* driven knockdown of *shopper* in the presence of an *Orco*^{dsRNA} dosis control construct shows a similar head bending phenotype. **e** *nrv2-Gal4* driven knockdown of *shopper* in the presence of the *D. virilis shopper* rescue construct shows a wild type locomotion pattern. **f** Quantification of head bending probability for the genotypes indicated. Note: overexpression of the *D.virilis* gene, a mock dsRNA construct as well as the co expression of the *shop*^{dsRNA} and the *D. virilis* contruct show significant lower head bending probability. See supplementary Table 1 for details on statistics.

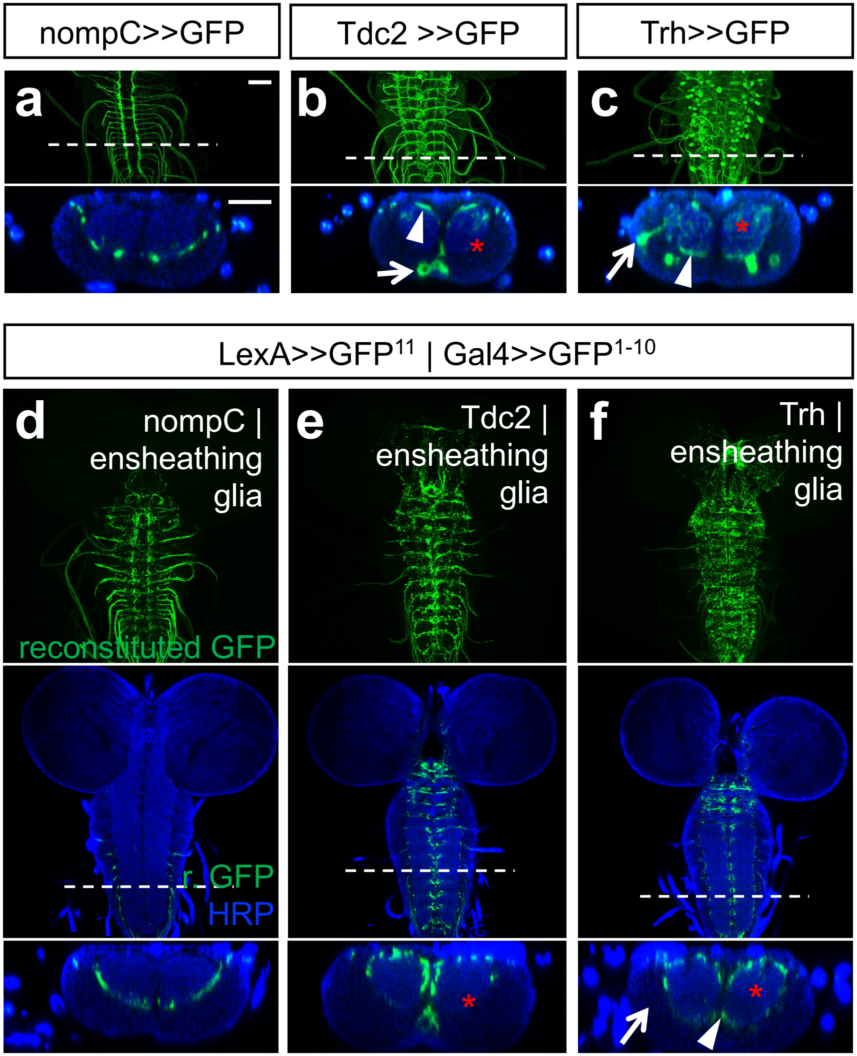

nompC>>GFP
Tdc2 >>GFP
Trh>>GFP
a
b
c
LexA>>GFP11 | Gal4>>GFP1-10
d
nompC | ensheathing glia
e
Tdc2 | ensheathing glia
f
Trh | ensheathing glia
reconstituted GFP
r. GFP
HRP

Supplementary Figure 4 Ensheathing glial cells contact selected axon tracts in the VNC neuropil

a-c The expression patterns of several *LexA* drivers are shown. *nompC-LexA* activates expression in sensory neurons projecting to the ventral side of the neuropil. The white dashed lines indicate the position of orthogonal sections shown below. **b** *Tdc2-LexA* activates expression in inter neurons (white arrow) projecting to the dorsal part of the neuropil (white arrow head) with some neurites within the neuropil (asterisk). **c** *Trh-LexA* activates expression in inter neurons (white arrow) projecting throughout the neuropil with many projections within the neuropil (red asterisk). **d** The interface of *nompC-LexA* expressing neurons and ensheathing glial cells is restricted to the ventral neuropil and the nerve roots. **e** The interface of *Tdc2-LexA* expressing neurons and ensheahting glial cells is restricted to the dorsal margin of the neuropil. Note that no reconstruction is found in the neuropil (asterisk). **f** The interface of *Trh-LexA* expressing neurons and ensheathing glial cells is restricted to the margin of the neruopil. Note that no reconstruction is visible in the neuropil (asterisk). Scale bars are 20 µm.

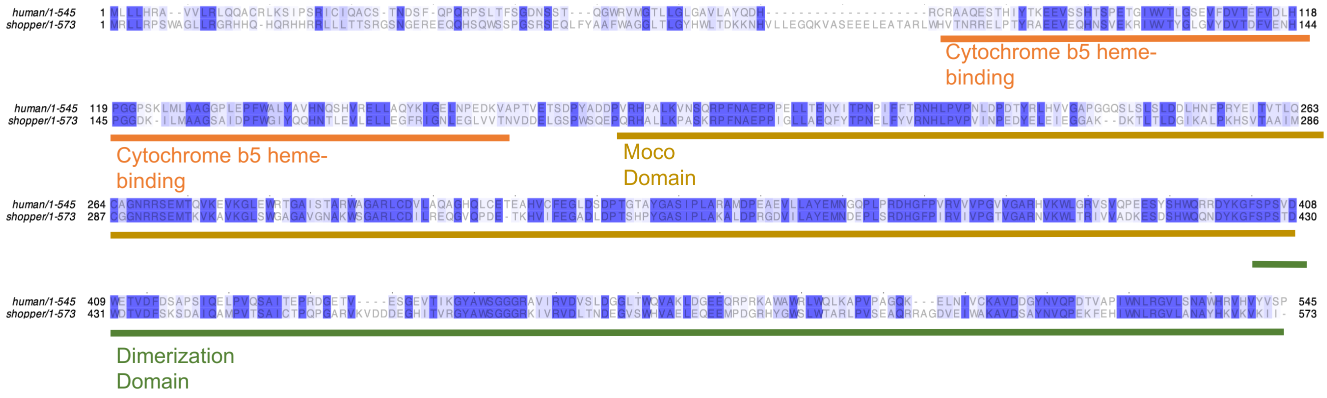

Supplementary Figure 5 Sequence comparison between Shopper and human Sulfite Oxidase

Amino acid sequences of Shopper are compared to the human SUOX. Identical amino acids are shaded in dark blue (52%), light blue shading indicates conserved amino acids, missing amino acids are marked by hyphens. The extents of functionally important domains are indicated. Sequence homology is high in all three, the Cytochrome b5 Hem-binding domain, the Moco binding domain, and the dimerization domain.

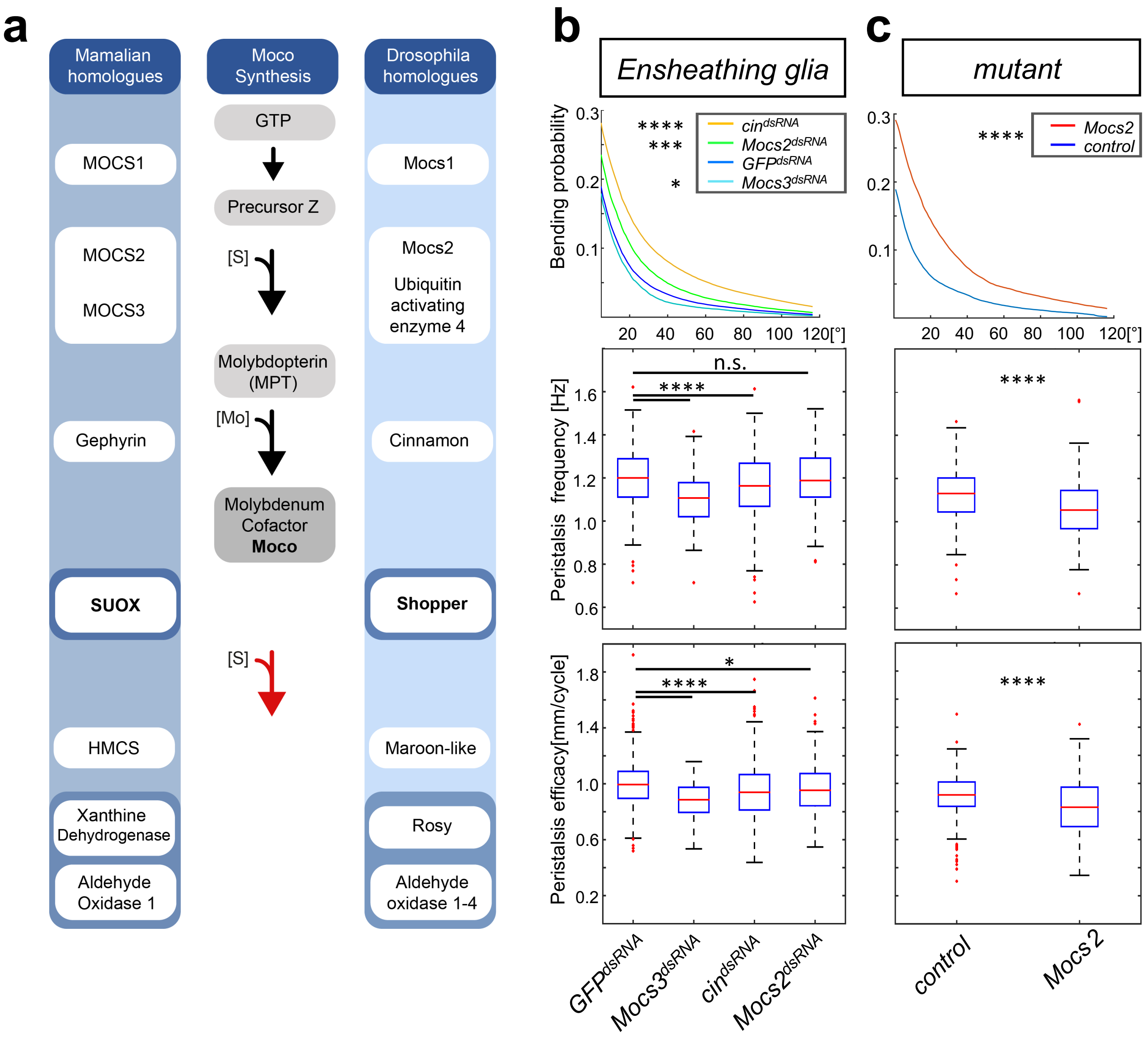

Supplementary Figure 6 Moco synthesis is required for larval locomotion control

a Comparison between mammalian and *Drosophila* Moco synthesis. The homologous gene names are noted at the relevant biochemical steps. For details see: (Marelja et al. 2018; Schwarz, 2016; Schwarz et al., 2009). **b** Functional analysis of enzymes involved in Moco synthesis. Suppression of *cinnamon* and *Mocs2* specifically in the ensheathing glial cells results in increased head bending. Suppression of *Mocs3* leads to aberrant peristalsis compared to controls. **c** A *Mocs2* mutant [*Mi{MIC}Mocs2MI07531*] compared to wild type controls shows a strongly increased head bending probability as well as reduced peristalsis frequency and efficacy similar to the phenotype observed upon lack of *shopper*. Box plots represent: The first and third quartile, as well as the median. Whiskers contain values within 1.5x of the interquartile range, outliers are red crosses. >600 frames of 50 - 100 larvae; *p<0.05, ***p<0.005, ****p<0.001, Wilcoxon rank-sum Test. See supplementary Table 1 for details.

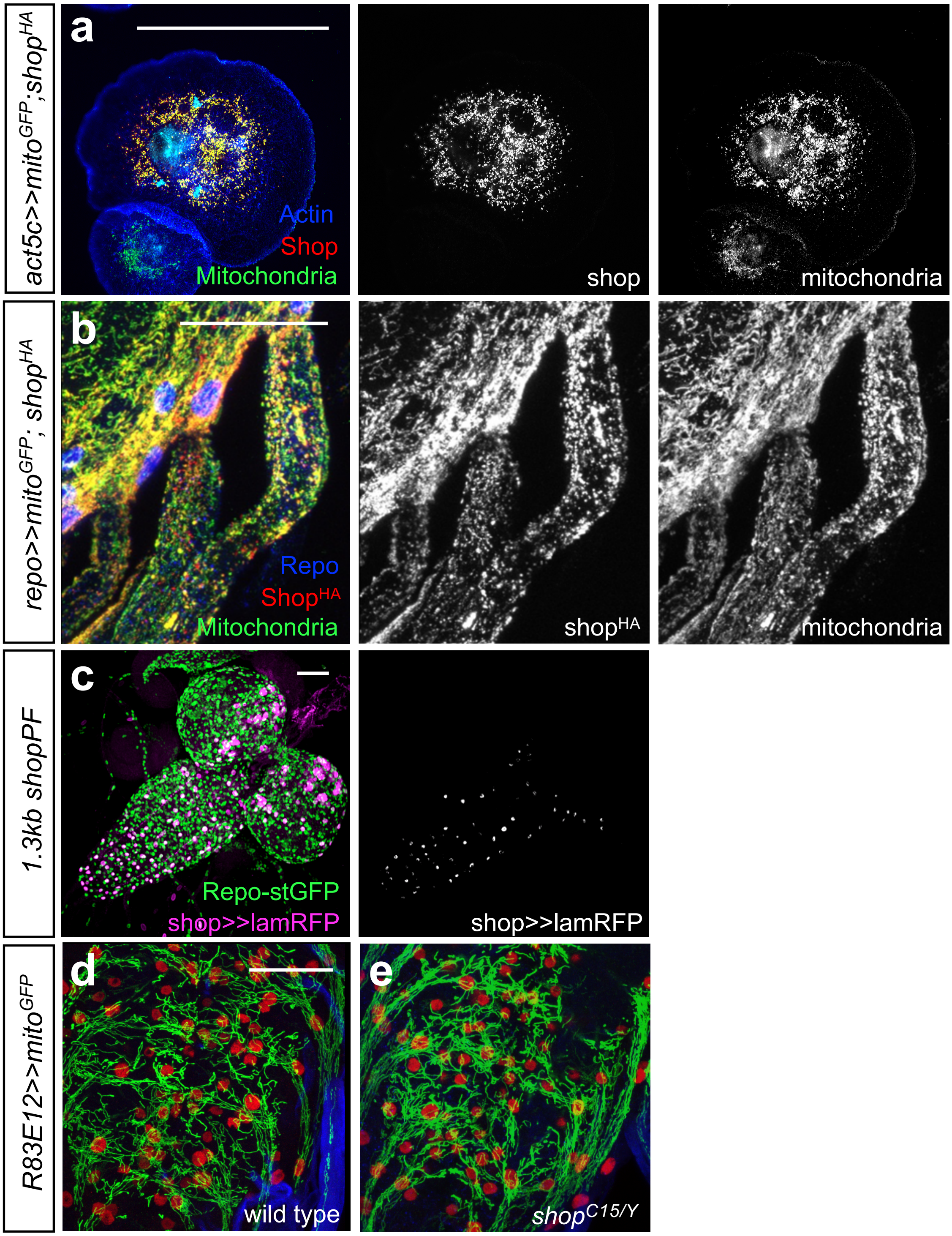

Supplementary Figure 7 Shopper localizes to glial mitochondria and *shop*^{C15} does not alter mitochondrial morphology

a *Drosophila* S2 tissue culture cells transfected with *UAS-mitoGFP* and *UAS-ShopHA*. HA expression (red) colocalizes with MitoGFP (green). Phalloidin staining (blue) indicates the F-actin cytoskeleton. **b** Dorsal view of a ventral nerve cord; genotype (*repo-Gal4, UAS-mitoGFP; UAS-shopperHA*). ShopperHA expression (red) is found in a dotted or tubular pattern corresponding to the GFP positive mitochondria (green). **c** The *1.3kb shopPF* promoter fragment directs expression of *Gal4* to drive *UAS-lamRFP* in a subset of glial cells that are labelled by a *repo-stingerGFP* expression construct. **d** Third instar larval brain expressing MitoGFP (green) in ensheathing glial cells. Glial nuclei are shown in red, neuronal membranes in blue (HRP staining). **e** *shopper* mutant third instar larval brain expressing MitoGFP (green) in ensheathing glial cells stained in **(d)**. No abnormal mitochondrial phenotype can be detected. Scale bars are 20 µm.

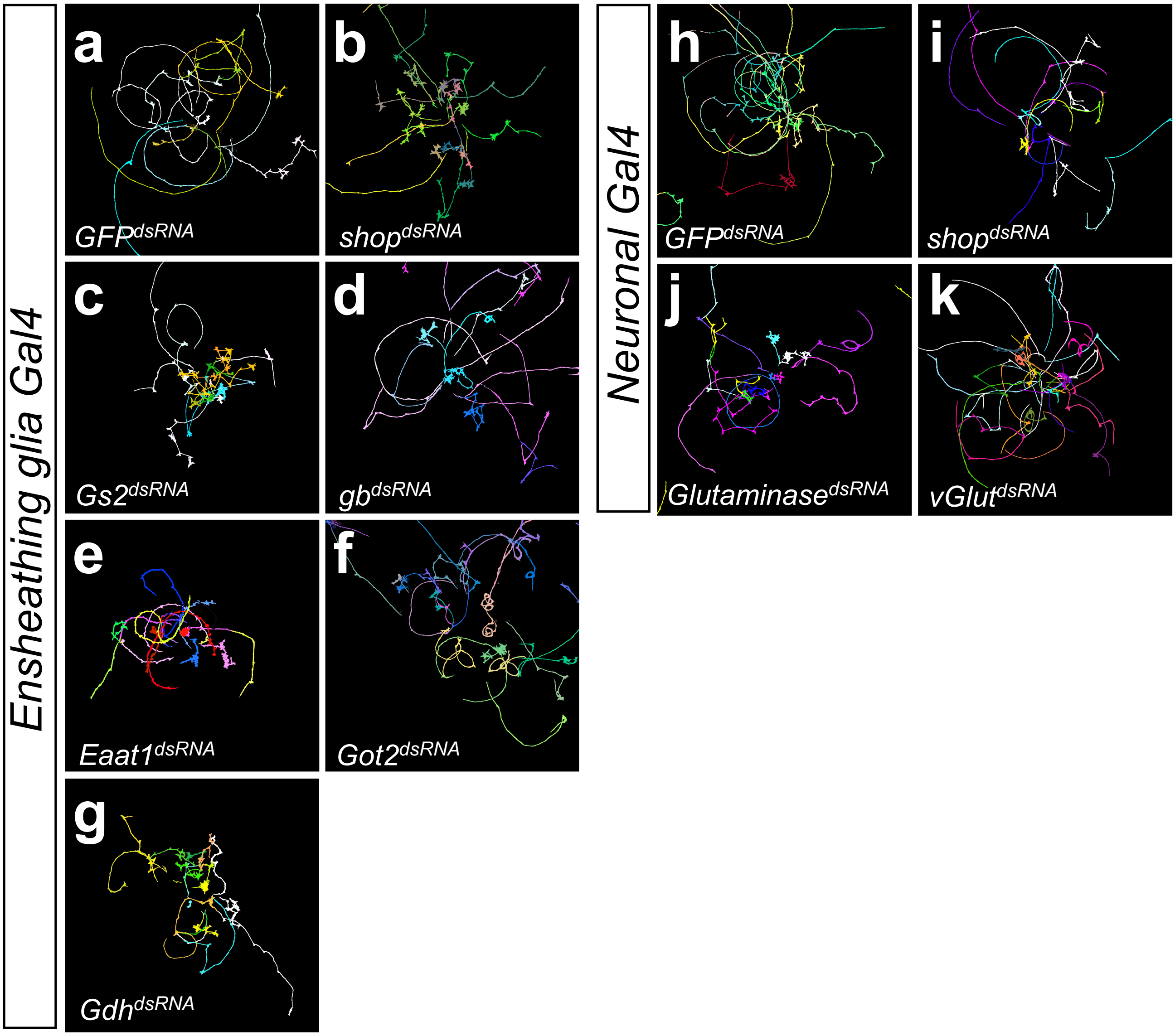

Supplementary Figure 8 Trajectories of larvae with impaired gene functions affecting the glutamate-glutamine cycle

Trajectories of ~15 larvae with different genes suppressed. The function of the following genes was suppressed in ensheathing glia (*R83E12-Gal4*) by expression of dsRNA constructs: *shopper, Eaat1, Gs2, gb, Got2, and Gdh. GFP*^{dsRNA} served as control. The following genes were tested in postmitotic neurons (*nsyb-Gal4*): *Glutaminase, vGlut*, and *shopper.* **a** *GFP*^{dsRNA} served as control. **b** Ensheathing glial specific knockdown of *shopper* causes aberrant locomotion with strong head bending. Knockdown of **c** *Eaat1*, **d** *Gs2*, **e** *gb*, **f** *Got2*, and **g** *Gdh* causes similar phenotypes with strong head bending. **h-g** Knockdown of genes affecting the glutamate- glutamine cycle in postmitotic neurons using *nsyb-Gal4*. **h** Neuronal expression of *GFP*^{dsRNA} caused no abnormal locomotion phenotype. Neuronal knockdown of *shopper* **i** caused no aberrant locomotion. Neuronal knockdown of *Glutaminase* **j** and *vGlut* **k** caused head bending phenotypes comparable to suppression of glutamate-glutamine cycle enzymes in glial cells.

Figure	Experiment	Combination	Summary	p/value	statistical test	n (t: trajectories; rp: run phases)
1,c	panglial bending probability	repo>GFPdsRNA vs. repo>shopdsRNA	****	6,90E-06	Wilcoxon rank-sum test	183; 190 t
1,d	panglial Distance to Origin	repo>GFPdsRNA vs. repo>shopdsRNA	****	8,60E-15	Wilcoxon rank-sum test	183; 190 t
1,e	panglial peristalsis frequency	repo>GFPdsRNA vs. repo>shopdsRNA	****	1,12E-13	Wilcoxon rank-sum test	483; 524 rp
1,f	panglial peristalsis efficiancy	repo>GFPdsRNA vs. repo>shopdsRNA	****	7,52E-07	Wilcoxon rank-sum test	483; 524 rp
1,k	ensheathing glia bending probability	R83E12>GFPdsRNA vs. R83E12>shopdsRNA	****	2,02E-06	Wilcoxon rank-sum test	329; 369 t
3,b	ensheathing glia cycle frequency	R83E12>GFPdsRNA vs. R83E12>shopdsRNA	*	0,0108	Mann-Whitney Rank Sum t	15; 11 animals
	ensheathing glia burst frequency	R83E12>GFPdsRNA vs. R83E12>shopdsRNA	n.s.	0,1090	Mann-Whitney Rank Sum t	15; 11 animals
	ensheathing glia interburst duration	R83E12>GFPdsRNA vs. R83E12>shopdsRNA	n.s.	0,0870	Mann-Whitney Rank Sum t	15; 11 animals
3,d	ensheathing glia cycle phase analysis	R83E12>GFPdsRNA vs. R83E12>shopdsRNA	*	0,0380	two tailed t-test	12; 7 animals
4,c	sulfite oxidase activity	w1118 vs. act>OrcodsRNA	n.s.	0,2398	two tailed t-test	3 measurements 3 experiments
		act>OrcodsRNA vs. act>shop dsRNA	***	0,0018		3 measurements 3 experiments
		w1118 vs. Df(1)shopper+4	***	0,0022		3 measurements 3 experiments
		act>OrcodsRNA vs. sopdsRNA; shopD.v.	**	0,0061		3 measurements 3 experiments
		w1118 vs. act>shopHA	*	0,0236		3 measurements 3 experiments
4,d	sulfite levels	w1118 vs. act>OrcodsRNA	n.s.	0,0904	two tailed t-test	3 measurements 3 experiments
		act>OrcodsRNA vs. act>shop dsRNA1	****	0,0000		3 measurements 3 experiments
		act>OrcodsRNA vs. act>shop dsRNA2	****	0,0000		3 measurements 3 experiments
		act>OrcodsRNA vs. sopdsRNA; shopD.v.	n.s.	0,1255		3 measurements 3 experiments
		w1118 vs. act>shopHA	*	0,0162		3 measurements 3 experiments
4g	shopper mutant bending probability	CRISPRcontrol vs. shopperC15	****	6,34E-12	Wilcoxon rank-sum test	162; 184 t
6a left	ensheathing glia glutamate metabolism bending probability	R83E12>GFPdsRNA vs. R83E12>shopdsRNA	****	2,02E-06	Wilcoxon rank-sum test	329; 369 t
		R83E12>GFPdsRNA vs. R83E12>EAAT1dsRNA	****	2,58E-18		329; 45 t
		R83E12>GFPdsRNA vs. R83E12>GDHdsRNA	****	4,50E-13		329; 71 t
		R83E12>GFPdsRNAvs. R83E12>GS2dsRNA	****	7,09E-14		329; 212 t
		R83E12>GFPdsRNA vs. R83E12>Got2dsRNA	****	5,30E-07		329; 137 t
		R83E12>GFPdsRNA vs. R83E12>gbdsRNA	****	5,98E-07		329; 102 t
6a right	pan neuronal glutamate metabolism bending probability	nsyb>GFPdsRNA vs. nsyb>shopdsRNA	n.s.	0,1736	Wilcoxon rank-sum test	378; 234 t
		nsyb>GFPdsRNA vs. nsyb>GlutaminasedsRNA	****	0,0001		378; 71 t
		nsyb>GFPdsRNA vs. nsyb>vGlutdsRNA	n.s.	0,1657		378; 340 t
6b left	ensheathing glia glutamate metabolism distance to origin	R83E12>GFPdsRNA vs. R83E12>shopdsRNA	*	0,0353	Wilcoxon rank-sum test	329; 369 t
		R83E12>GFPdsRNA vs. R83E12>EAAT1dsRNA	****	8,29E-11		329; 45 t
		R83E12>GFPdsRNA vs. R83E12>GDHdsRNA	****	7,72E-11		329; 71 t
		R83E12>GFPdsRNAvs. R83E12>GS2dsRNA	****	2,88E-05		329; 212 t
		R83E12>GFPdsRNA vs. R83E12>Got2dsRNA	n.s.	0,1332		329; 137 t
		R83E12>GFPdsRNA vs. R83E12>gbdsRNA	n.s.	0,1240		329; 102 t
6b right	pan neuronal glutamate metabolism distance to origin	nsyb>GFPdsRNA vs. nsyb>shopdsRNA	n.s.	0,1020	Wilcoxon rank-sum test	378; 234 t
		nsyb>GFPdsRNA vs. nsyb>GlutaminasedsRNA	*	0,0194		378; 71 t
		nsyb>GFPdsRNA vs. nsyb>vGlutdsRNA	**	0,0054		378; 340 t
6c left	ensheathing glia glutamate metabolism peristalsis frequency	R83E12>GFPdsRNA vs. R83E12>shopdsRNA	****	2,62E-18	Wilcoxon rank-sum test	982; 1097 rp
		R83E12>GFPdsRNA vs. R83E12>EAAT1dsRNA	****	2,47E-35		982; 232 rp
		R83E12>GFPdsRNA vs. R83E12>GDHdsRNA	****	3,52E-18		982; 351 rp
		R83E12>GFPdsRNAvs. R83E12>GS2dsRNA	****	2,64E-05		982; 651 rp
		R83E12>GFPdsRNA vs. R83E12>Got2dsRNA	****	1,86E-11		982; 137 rp
		R83E12>GFPdsRNA vs. R83E12>gbdsRNA	**	0,0073		982; 306 rp
6c right	pan neuronal glutamate metabolism peristalsis frequency	nsyb>GFPdsRNA vs. nsyb>shopdsRNA	n.s.	0,3468	Wilcoxon rank-sum test	857; 428 rp
		nsyb>GFPdsRNA vs. nsyb>GlutaminasedsRNA	****	3,22E-11		857; 222 rp
		nsyb>GFPdsRNA vs. nsyb>vGlutdsRNA	****	3,16E-08		857; 764 rp
6d left	ensheathing glia glutamate metabolism peristalsis effciacy	R83E12>GFPdsRNA vs. R83E12>shopdsRNA	****	3,02E-09	Wilcoxon rank-sum test	982; 1097 rp
		R83E12>GFPdsRNA vs. R83E12>EAAT1dsRNA	****	7,36E-26		982; 232 rp
		R83E12>GFPdsRNA vs. R83E12>GDHdsRNA	****	1,08E-43		982; 351 rp
		R83E12>GFPdsRNAvs. R83E12>GS2dsRNA	n.s.	0,1479		982; 651 rp
		R83E12>GFPdsRNA vs. R83E12>Got2dsRNA	****	0,0002		982; 137 rp
		R83E12>GFPdsRNA vs. R83E12>gbdsRNA	n.s.	0,5328		982; 306 rp
6d right	pan neuronal glutamate metabolism peristalsis efficiency	nsyb>GFPdsRNA vs. nsyb>shopdsRNA	*	0,0254	Wilcoxon rank-sum test	857; 428 rp
		nsyb>GFPdsRNA vs. nsyb>GlutaminasedsRNA	***	0,0039		857; 222 rp
		nsyb>GFPdsRNA vs. nsyb>vGlutdsRNA	****	2,68E-05		857; 764 rp
7,b upper	ubiquitious GDH expression rescue peristalsis frequency	shopC15; act> w1118 vs. shopC15; act> GDH	****	0,0001	Wilcoxon rank-sum test	140; 223 rp
	ubiquitious GDH expression rescue peristalsis efficacy	shopC15; act> w1118 vs. shopC15; act> GDH	n.s.	0,0576		140; 223 rp
	ubiquitious GDH expression rescue bending probability	shopC15; act> w1118 vs. shopC15; act> GDH	****	0,0007		16; 29 t
7,b middle	ensheathing glial GDH expression rescue	shopC15; R83E12> w1118 vs. shopC15; R83E12> GDH	****	8,39E-09	Wilcoxon rank-sum test	245; 445 rp
	ensheathing glial GDH expression rescue peristalsis efficacy	shopC15; R83E12> w1118 vs. shopC15; R83E12> GDH	n.s.	0,7976		245; 445 rp
	ensheathing glial GDH expression rescue bending probability	shopC15; R83E12> w1118 vs. shopC15; R83E12> GDH	*	0,0115		26; 46 t
7,b lower	Cortex glial GDH expression rescue peristalsis frequency	shopC15; R55B12> w1118 vs. shopC15; R55B12> GDH	n.s.	0,1332	Wilcoxon rank-sum test	171; 116 rp
	Cortex glial GDH expression rescue peristalsis efficacy	shopC15; R55B12> w1118 vs. shopC15; R55B12> GDH	*	0,0424		171; 116 rp
	Cortex glial GDH expression rescue bending probability	shopC15; R55B12> w1118 vs. shopC15; R55B12> GDH	n.s.	0,9727		21; 20 t
S2,e	Cortex glia bending probability	R55B12 > GFPdsRNA vs. R55B12 > shopdsRNA	n.s.	0,7834	Wilcoxon rank-sum test	253; 206 t
	Astroyte like glia bending probability	alrm > GFPdsRNA vs. alrm > shopdsRNA	n.s.	0,5384		116; 145 t
S2,h	R83E12 in 86Fb bending probability	R83B12_86Fb > GFPdsRNA vs. R83E12_86Fb > shopdsRNA	****	2,70E-21	Wilcoxon rank-sum test	87; 125 t
S2,i	R83E12 in 86Fb temperature dependency	R83B12_86Fb > shopdsRNA_19C° vs. R83E12_86Fb_25° > shopdsRNA	****	6,98E-21	Wilcoxon rank-sum test	74; 125 t
S2,j	R25H07 in attP2 bending probability	R25H07_attP2 > GFPdsRNA vs. R25H07_attP2 > shopdsRNA	n.s.	0,6021	Wilcoxon rank-sum test	87; 79 t
	R25H07 in attP2 peristalsis frequency	R25H07_attP2 > GFPdsRNA vs. R25H07_attP2 > shopdsRNA	n.s.	0,7752	Wilcoxon rank-sum test	288; 459 rp
	R25H07 in attP2 peristalsis efficiency	R25H07_attP2 > GFPdsRNA vs. R25H07_attP2 > shopdsRNA	****	3,47E-08	Wilcoxon rank-sum test	288; 459 rp
S3,f	Interspecies Rescue bending probability	nrv2 > OrcodsRNA vs. nrv2 > shopdsRNA	****	2,27E-12	Wilcoxon rank-sum test	224; 263 t
		nrv2 > shopdsRNA vs. nrv2 > shopdsRNA, shopD.v.	****	6,64E-08		224; 350 t
		nrv2 > shopD.v. vs. nrv2 > shopdsRNA, shopD.v.	n.s.	0,6296		171; 350 t
		nrv2 > OrcodsRNA vs. nrv2 > shopdsRNA, orcodsRNA	****	1,75E-08		224; 41 t
		nrv2 > OrcodsRNA vs. nrv2 > shopD.v.	n.s.	0,0882		224; 171 t
		nrv2 > OrcodsRNA vs. nrv2 > shopdsRNA; shopD.v.	*	0,0273		224; 350 t
S6, b upper	Moco synthesis ensheathing glia bending probability	R83B12 > GFPdsRNA vs. R83E12 > Mocs3dsRNA	*	0,0173	Wilcoxon rank-sum test	150; 40 t
		R83B12 > GFPdsRNA vs. R83E12 > Mocs2dsRNA	***	0,0039		150; 183 t
		R83B12 > GFPdsRNA vs. R83E12 > cindsRNA	****	1,40E-11		150; 41 t
S6, c upper	Moco synthesis in mutant bending probability	w1118 vs. *mocs2*	****	3,80E-11	Wilcoxon rank-sum test	167; 55 t
S6, b middle	Moco synthesis ensheathing glia peristalsis frequency	R83B12 > GFPdsRNA vs. R83E12 > Mocs3dsRNA	****	4,13E-13	Wilcoxon rank-sum test	467; 113 rp
		R83B12 > GFPdsRNA vs. R83E12 > Mocs2dsRNA	n.s.	0,9030		467; 748 rp
		R83B12 > GFPdsRNA vs. R83E12 > cindsRNA	****	0,0001		467; 130 rp
S6, c middle	Moco synthesis in mutant peristalsis frequency	w1118 vs. *mocs2*	****	2,37E-07	Wilcoxon rank-sum test	327; 162 rp
S6, b lower	Moco synthesis ensheathing glia peristalsis efficacy	R83B12 > GFPdsRNA vs. R83E12 > Mocs3dsRNA	****	4,86E-12	Wilcoxon rank-sum test	467; 113 rp
		R83B12 > GFPdsRNA vs. R83E12 > Mocs2dsRNA	*	0,0231		467; 748 rp
		R83B12 > GFPdsRNA vs. R83E12 > cindsRNA	****	9,46E-07		467; 130 rp
S6, c lower	Moco synthesis in mutant peristalsis efficacy	w1118 vs. *mocs2*	****	1,41E-05	Wilcoxon rank-sum test	327; 162 rp

Supplementary Table 1 Statistical analyses

Summary of all statistical analyses conducted in this paper, grouped according to the figures. The genotypes used in the different experiments, the n-numbers, the statistical tests, and the p-values are indicated. In general, asterisk indicate p-values of: *<0.05, **<0.01, ***<0.005, ****<0.001.

Supplementary References:

Schwarz, Günter, 2016. Molybdenum cofactor and human disease. Current Opinion in Chemical Biology 31, 179–187. doi:10.1016/j.cbpa.2016.03.016

Schwarz, Günter, Mendel, R.R., Ribbe, M.W., 2009. Molybdenum cofactors, enzymes and pathways. Nature 460, 839–847. doi:10.1038/nature08302

Marelja, Z., Leimkühler, S., Missirlis, F., 2018. Iron Sulfur and Molybdenum Cofactor Enzymes Regulate the Drosophila Life Cycle by Controlling Cell Metabolism. Frontiers in physiology 9, 50. doi:10.3389/fphys.2018.00050
